# Prevalence and factors associated with active transportation to school for adolescents

**DOI:** 10.11606/s1518-8787.2020054002078

**Published:** 2020-08-05

**Authors:** Edina Maria de Camargo, Michael Pereira da Silva, Jorge Mota, Wagner de Campos

**Affiliations:** I Universidade Federal do Paraná Programa de Pós-Graduação em Educação Física CuritibaPR Brasil Universidade Federal do Paraná. Programa de Pós-Graduação em Educação Física. Curitiba, PR, Brasil; II Universidade Federal do Rio Grande Faculdade de Medicina Rio GrandeRS Brasil Universidade Federal do Rio Grande. Faculdade de Medicina. Rio Grande, RS, Brasil; III Universidade do Porto Faculdade de Desporto da Universidade do Porto Departamento de Educação Física Porto Portugal Universidade do Porto. Faculdade de Desporto da Universidade do Porto. Departamento de Educação Física. Porto, Portugal; IV Universidade Federal do Paraná Departamento de Educação Física CuritibaPR Brasil Universidade Federal do Paraná. Departamento de Educação Física. Curitiba, PR, Brasil

**Keywords:** Adolescent, Walking, Motor Activity, Socioeconomic Factors, Cross-Sectional Studies

## Abstract

**OBJECTIVE:**

To verify the prevalence and factors associated with active transportation to school (ATS) among Brazilian adolescents attending public schools.

**METHODS:**

Crossectional study with a representative sample of 1,984 adolescents (55.9% girls). Sociodemographic variables included were: gender, age, parental schooling, and socioeconomic status. Psychosocial factors included were: social support from parents and friends for physical activity. Walking, cycling, or skateboarding to school were considered models of active transportation. Binary logistic regression models verified sociodemographic and psychosocial factors association with ATS, adopting p < 0.05.

**RESULTS:**

The prevalence of active transportation to school was 37.7% (16.2% boys and 21.5% girls). For boys, ATS was associated with: social support from parents in practicing physical activity together as a family (OR = 1.57; 95%CI 1.09–2.25), giving them rides (OR = 1.56; 95%CI 1.04–2.32), and remarking their good performance on it (OR = 1.73; CI95 1.08–2.76); as well as the social support from friends in practicing physical activity together (OR = 2.23; 95%CI 1.35–3.69). For girls, the likelihood of using ATS increased with age (OR = 1.43; 95%CI 1.06–1.92) and having friends who practice physical activity together with them (OR = 1.48, 95%CI 1.04–2.10).

**CONCLUSION:**

Age and social support for physical activity were associated with ATS. Parents who practice together, give rides, and remark on physical activities increase the likelihood of adolescent boys using ATS. Social support from friends to physical activity increased the likelihood of both genders using ATS.

## INTRODUCTION

Active transportation to school (ATS) is an effective approach to improve overall physical activity (PA) levels among adolescents^1^. Adolescents involved in ATS, such as walking and cycling, accumulate more PA and have lower cardiometabolic risks such as overweight and obesity, diabetes, and metabolic syndrome^2^^–^^4^. Despite the potential health benefits of ATS, studies have reported a drop in the proportion of children and adolescents using it in recent decades^2^^–^^4^, which may contribute to a global decline in PA levels. Thus, promoting ATS has featured in international initiatives aimed at increasing PA at the population level^3^^–^^6^.

Ecological models state that health behaviors, such as active transportation, are influenced by various factors at multiple levels, including psychosocial and environmental factors^5^^,^^6^. Such factors may vary according to gender, age, parental schooling, and socioeconomic status^5^^,^^6^. As an example, adolescents living near school are more likely to use ATS^1^^,^^2^; however, older adolescents often use passive transportation, such as car and motorcycle, even to nearby destinations^1^^,^^2^. These results suggest a need for greater efforts to understand factors that may influence the use of ATS, aiming to increase the use of this mode of commuting to school and other destinations^1^^,^^2^.

In recent years, Brazil have attempted to improve the urban environment by creating bike paths and bike lanes, which may favor the use of active transport modes by the population.^7^ This type of initiative have a medium- to long-term impact, as well as a wide reach^8^. Understanding the relation between sociodemographic and psychosocial factors – specifically the support from parents and friends to practice physical activity in different contexts (leisure and transportation) – may provide valuable information to public health authorities in implementing and promoting ATS and, consequently, improving PA levels. This study aimed to investigate the prevalence and factors associated with active transportation to school among Brazilian adolescents attending public schools.

## METHODS

This is a cross-sectional study conducted in 2018, with a representative sample of adolescents aged from 15 to 17 years, attending public high schools in Curitiba, in the state of Paraná, Brazil. This study followed the recommendations of the National Health Council (Resolution No. 466/2012) for research involving human beings, and it was approved by the Research Ethics Committee of the Universidade Federal do Paraná (CAAE: 98133218.8.0000.0102) and authorized by adolescents’ parents or legal guardians upon signing the informed consent form.

According to the 2017 school census, conducted by the National Institute for Educational Studies and Research Anísio Teixeira (INEP), the public schools of the city had 53,760 adolescents, of both genders and aged from 15 to 17 years, enrolled in high school. G*Power was used to estimate the sample. We adopted a 1.49 prevalence ratio (PR) between social support and physical activity^9^, 50% prevalence of active transport^2^, 95% confidence level (α = 0.05) with 80% power (β = 0.20), and a 30% increase to compensate possible losses and refusals. Estimated necessary sample size was 1,930 adolescents: 965 boys and 965 girls.

Sampling procedure was initiated by conglomerates, in three stages. First, all public schools within each of the nine administrative regions of Curitiba were stratified; second, two schools of each region were drawn; third, one class of each high school year was randomly selected, considering the number of students required by gender for a given administrative region of the municipality. The random selection contemplated both morning and afternoon classes. All students from the selected classes were invited to participate in the study.

The total of 2,506 adolescents were invited to participate in the study. Those who failed in presenting the informed consent form signed by a parent or legal guardian (n = 100), those who refused to participate in the study, and those who were absent on the collection day (n = 56) were not included. We excluded those who reported physical or cognitive limitations associated with PA practice (n = 12) and those aged 18 years (n = 125). Adolescents who answered the questionnaires incorrectly (n = 229) were considered sample loss. 1,984 adolescents composed the study analytical sample. A posteriori power analyses showed that this sample could identify statistically significant prevalence ratios above OR = 1.28 as an increase in the use of active transportation to school, and below OR = 0.77 as lower likelihood in using active transportation to school, considering a 34% prevalence of adolescents with poor social support and without the habit of using active transportation.

### Gender, Age, Schooling, and Socioeconomic Status

Gender was self-reported (male or female) and age was estimated from the date of birth informed by the adolescent and classified into 15, 16, or 17 years. Parental schooling, as well as socioeconomic status, were classified according to the Brazilian Association of Research Companies (BARC)^10^, into the following categories: elementary school, high school, or college (we asked for father and mother's schooling and the head of the household). The Brazilian Criteria of Economic Classification (BCEC)^10^ classifies the social strata into the economic classes A, B1, B2, C1, C2, and D-E, based on data from the National Household Sample Survey (NHSS). For analysis, and aiming to ensure comparability to related studies, socioeconomic status (SES) were classified into three categories: lower (classes C+D+E), middle (B1+B2), and higher (A1+A2).

### Social Support from Parents and Friends

Social support from parents and friends for PA practice was measured using a 10-item scale – the ASAFA Scale, – which presents satisfactory internal consistency (parents: α ≥ 0.77 and composite reliability index [CRI] ≥ 0.83; friends: α ≥ 0.87 and CRI ≥ 0.91)^11^. The adolescents reported the frequency (never = 1, rarely = 2, often = 3, always = 4) with which parents and friends offered some kind of social support for PA practice (encourage, practice, ride, assist, remark, invite) during a typical week^11^, by answering the questions: “How often do your parents: Encourage you to practice PA? Practice PA with you? Give you a ride or provide transportation for you to go to the place where you practice PA? Watch you practicing PA? Remark your good performance in the PA?” and “How often do your friends: Encourage you to practice PA? Practice PA with you? Invite you to practice PA with them? Watch you practicing PA? Remark your good performance in the PA?”

For analysis, and aiming to ensure comparability to related studies^12^^,^^13^, “rarely” and “frequently” were grouped and classified as “sometimes.” Weekly frequency of PA was classified as never, sometimes or always.

### Active transportation to school

We assessed the used mode of transportation to and from school during a typical week (walking, biking, skateboarding, bus, van, or car). Students who reported walking, cycling, or skateboarding to and from school were considered “active”; the others were considered “passive.” This question presented adequate test-retest reliability (intraclass correlation coefficient 0.90–0.95; p < 0.05) and has been applied by related studies^14^^,^^15^.

### Data Analysis

To avoid bias related to the complex sampling process (stratified cluster), association analyses were corrected by the complex delineation, using the complex sample command in the SPSS Statics 23.0. Such procedure was adopted to ensure that estimates would reflect population data from the elementary sampling units.

Prevalence was described by relative and absolute frequency distribution. Pearson's chi-square test was used to compare proportions between genders; then a post hoc test was used to demonstrate which categories showed the greatest difference. The crude and adjusted binary logistic regression was applied to verify the association of demographic factors and social support from parents and friends for ATS. Following the stepwise criterion, p-value ≤ 0.20 was adopted for input variables in the fitting model. All analyses were performed separately for each gender; 5% significance level was adopted.

## RESULTS

The sample was composed of 1,984 adolescents, 55.9% of which were female. Among these, 748 (37.7%) only reported using active transportation to school in a typical week: 16.2% boys and 21.5% girls. Tables 1 and 2 show the data stratified by gender.

**Table 1:** Adolescents’ age, parental schooling, and socioeconomic status (SES) according to gender (n = 1,984).

		Male (n = 875; 44.1%)		Female (n = 1,109; 55.9%)			Total	
		n	%	n	%	p^c^	n	%
Age	15 years	261	41.8	363	58.2	0.124	624	100
	16 years	317	44.3	399	55.7		716	
	17 years	297	46.1	347	53.9		644	
Father's schooling	Elementary school	238^a^	38.1	387^a^	61.9	0.003	625	100
	High school	405^b^	47.2	453^b^	52.8		858	
	College	232^b^	46.3	269^b^	53.7		501	
Mother's schooling	Elementary school	245^a^	39.0	384^a^	61.0	0.006	629	100
	High school	398^b^	46.3	461^b^	53.7		859	
	College	232^b^	46.8	264^b^	53.2		496	
SES	Lower	140	40.0	210	60.0	0.005	350	100
	Middle	538^a^, ^b^	43.4	702^a^, ^b^	56.6		1,240	
	Higher	197	50.0	197	50.0		394	

a,b Significantly differ from each other; post hoc of Bonferroni.

c Chi-square test.

**Table 2:** Prevalence of social support from parents and friends among adolescents according to gender (n = 1,984).

			Male (n = 875; 44.1%)		Female (n = 1,109; 55.9%)			Total	
			n	%	n	%	p^c^	n	%
Social support from parents									
	Encourage	Never	170	41.5	240	58.5	0.113	410	100
		Sometimes	473	43.9	604	56.1		1,077	
		Always	232	46.7	265	53.3		497	
	Practice	Never	322	41.4	455	58.6	0.027	777	100
		Sometimes	434	45.4	530	55.0		964	
		Always	119	49.0	124	51.0		243	
	Give a ride (or provide transportation)	Never	425	44.0	541	56.0	0.513	966	100
		Sometimes	262	42.6	353	57.4		615	
		Always	188	46.7	215	53.3		403	
	Watch	Never	370	42.5	500	57.5	0.166	870	100
		Sometimes	365	44.8	450	55.2		815	
		Always	140	46.8	159	53.2		299	
	Remark	Never	324	41.9	449	58.1	0.621	773	100
		Sometimes	341	47.6	375	52.4		716	
		Always	210	42.4	285	57.6		495	
Social support from friends									
	Encourage	Never	324	42.1	445	57.9	0.240	769	100
		Sometimes	355	45.5	426	54.5		781	
		Always	196	45.2	238	54.8		434	
	Practice	Never	229^a^	39.7	348^a^	60.3	0.005	577	100
		Sometimes	370^a^,^b^	44.6	460^a^,^b^	55.4		830	
		Always	276^b^	47.8	301^b^	52.2		577	
	Invite	Never	243^a^	39.7	376^a^	60.7	0.015	619	100
		Sometimes	381^b^	46.4	440^b^	53.6		821	
		Always	251^a^,^b^	46.1	293^a^,^b^	53.9		544	
	Watch	Never	421	44.7	520	55.3	0.811	941	100
		Sometimes	309	43.0	410	57.0		719	
		Always	145	44.8	179	55.2		324	
	Remark	Never	425	42.9	566	57.1	0.462	991	100
		Sometimes	308	45.9	363	54.1		671	
		Always	142	44.1	180	55.9		322	

a,b Significantly differ from each other; post hoc of Bonferroni.

c Chi-square test.

Table 3 shows the association between sociodemographic factors and social support for active transportation to school (ATS) among boys. Regarding sociodemographic factors, we found no association between sociodemographic variables and ATS. As for social support from parents, results obtained by the adjusted analysis show that boys whose parents encourage physical activity (PA) practice were less likely to use active transportation (sometimes: odds ratio [OR] = 0.64; 95%CI 0.42–0.96; always: OR = 0.58; 95%CI 0.36–0.95). Conversely, boys were more likely to use ATS when parents sometimes practice PA with them (OR = 1.57; 95%CI 1.09–2.25), always offer transportation for the PA practice (OR = 1.56; 95%CI 1.04–2.32), and always remark their good performance in the PA (OR = 1.73; 95%CI 1.08–2.76).

**Table 3:** Crude and adjusted association of sociodemographic factors and social support from parents and friends for active transportation to school among boys (n = 875).

Sociodemographic factors		Crude					Adjusted		
		n	%	OR	95%CI	p	OR	95%CI	p
Age	15 years	98	44.3	1.00					
	16 years	129	43.1	1.14	0.81–1.60	0.441			
	17 years	95	41.7	0.78	0.55–1.11	0.169			
Father's schooling	Elementary school	151	38.8	1					
	High school	251	47.8	1.13	0.79–1.61	0.508			
	College	151	46.9	1.02	0.67–1.55	0.924			
Mother's schooling	Elementary school	96	37.6	1.00					
	High school	146	45.8	0.87	0.61–1.23	0.422			
	College	80	46.0	0.81	0.54–1.23	0.321			
SES	Lower	49	37.4	1.00					
	Middle	192	42.0	1.03	0.70–1.52	0.880			
	Higher	81	50.6	1.30	0.83–2.03	0.256			

Social support from parents		n	%	OR	95%CI	p	OR	95%CI	p
Encourage	Never	67	38.7	1.00			1.00		
	Sometimes	169	43.4	0.67	0.45–1.00	0.051	0.64	0.42–0.96	0.033
	Always	86	46.2	0.60	0.37–0.98	0.039	0.58	0.36–0.95	0.030
Practice	Never	99	35.7	1.00			1.00		
	Sometimes	178	46.8	1.49	1.05–2.10	0.026	1.57	1.09–2.25	0.015
	Always	45	49.5	1.19	0.70–2.03	0.522	1.23	0.72–2.09	0.443
Give a ride (or provide transportation)	Never	140	38.5	1.00			1.00		
	Sometimes	95	42.4	1.06	0.75–1.51	0.735	1.03	0.72–1.48	0.869
	Always	87	54.4	1.64	1.11–2.41	0.013	1.56	1.04–2.32	0.030
Watch	Never	130	40.5	1.00					
	Sometimes	139	43.6	0.83	0.57–1.21	0.322			
	Always	53	49.1	0.74	0.44–1.23	0.247			
Remark	Never	99	36.4	1.00			1.00		
	Sometimes	134	47.5	1.55	1.04–2.32	0.031	1.39	0.93–2.05	0.105
	Always	89	45.9	1.72	1.09–2.73	0.020	1.73	1.08–2.76	0.023

Social support from friends		n	%	OR	95%CI	p	OR	95%CI	p
Encourage	Never	117	40.1	1.00					
	Sometimes	137	46.3	0.93	0.64–1.34	0.687			
	Always	68	42.5	0.65	0.40–1.06	0.082			
Practice	Never	70	34.8	1.00			1.00		
	Sometimes	140	43.5	1.54	1.00–2.39	0.050	1.50	0.97–2.30	0.066
	Always	112	49.8	1.97	1.16–3.33	0.011	2.23	1.35–3.69	0.002
Invite	Never	81	33.8	1.00					
	Sometimes	138	46.8	1.03	0.65–1.58	0.954			
	Always	103	48.4	1.40	0.82–2.38	0.222			
Watch	Never	157	43.7	1.00			1.00		
	Sometimes	115	42.4	0.70	0.47–1.04	0.076	0.63	0.42–0.95	0.026
	Always	50	42.4	0.53	0.30–0.93	0.026	0.45	0.25–0.79	0.006
Remark	Never	146	39.7	1.00					
	Sometimes	122	46.7	1.30	0.90–1.88	0.165			
	Always	54	45.4	1.31	0.78–2.19	0.304			

OR: odds ratio; 95%CI: 95% confidence interval

Note: Only the variables presenting p values ≤ 0.20 in the crude analysis remained in the adjusted analysis.

Regarding social support from friends, boys who reported having friends that always practice PA were more likely to use ATS (OR = 2.23; 95%CI 1.35–3.69). Boys who have friends that watch them performing PA were less likely to use ATS (sometimes: OR = 0.63; 95%CI 0.42–0.95; always: OR = 0.45; 95%CI 0.25–0.79).

Table 4 shows the association between sociodemographic factors and social support for ATS for boys. Among the evaluated sociodemographic variables, only age was significant in the adjusted model – girls aged 16 years were more likely to use ATS (OR = 1.43; 95%CI 1.06–1.92). Regarding variables related to social support (SS) for PA, only those referring to SS from friends remained associated in the adjusted model – girls who have friends who sometimes practice PA together with them are more likely to use ATS than those who never get this type of support (OR = 1.48; 95%CI 1.04–2.10). Conversely, girls who have friends who sometimes invite them to practice PA were less likely to use ATS (OR = 0.65; 95%CI 0.46–0.93).

**Table 4:** Crude and adjusted association of sociodemographic factors and social support from parents and friends for active transportation to school among girls (n = 1,109).

Sociodemographic factors		Crude					Adjusted		
		n	%	OR	95%CI	p	OR	95%CI	p
Age	15 years	123	55.7	1.00			1.00		
	16 years	170	56.9	1.45	1.08–1.95	0.014	1.43	1.06–1.92	0.018
	17 years	133	58.3	1.21	0.89–1.65	0.218	1.17	0.86–1.60	0.317
Father's schooling	Elementary school	238	61.2	1.00					
	High school	274	52.2	1.13	0.84–1.53	0.418			
	College	171	53.1	1.03	0.71–1.48	0.854			
Mother's schooling	Elementary school	159	62.4	1.00					
	High school	173	54.2	0.82	0.60–1.10	0.182			
	College	94	54.0	0.77	0.54–1.11	0.156			
SES	Lower	82	62.6	1.00					
	Middle	265	58.0	0.95	0.70–1.30	0.734			
	Higher	79	49.4	1.05	0.70–1.56	0.828			

Social support from parents		n	%	OR	95%CI	p	OR	95%CI	p
Encourage	Never	106	61.3	1.00					
	Sometimes	220	56.6	0.70	0.50–0.98	0.040			
	Always	100	53.8	0.80	0.52–1.23	0.314			
Practice	Never	178	64.3	1.00					
	Sometimes	202	53.2	1.04	0.77–1.40	0.809			
	Always	46	50.5	1.03	0.63–1.66	0.921			
Give a ride (or provide transportation)	Never	224	61.5	1.00					
	Sometimes	129	57.6	0.79	0.58–1.07	0.124			
	Always	73	45.6	0.71	0.48–1.04	0.076			
Watch	Never	191	59.5	1.00					
	Sometimes	180	56.4	1.20	0.87–1.66	0.258			
	Always	55	50.9	0.98	0.62–1.54	0.919			
Remark	Never	173	63.6	1.00					
	Sometimes	148	52.5	1.18	0.84–1.66	0.340			
	Always	105	54.1	1.08	0.72–1.61	0.722			

Social support from friends		n	%	OR	95%CI	p	OR	95%CI	p
Encourage	Never	175	59.9	1.00					
	Sometimes	159	53.7	0.98	0.70–1.37	0.908			
	Always	92	57.5	1.09	0.72–1.66	0.683			
Practice	Never	131	65.2	1.00			1.00		
	Sometimes	182	56.5	1.39	0.96–2.01	0.084	1.48	1.04–2.10	0.030
	Always	113	50.2	1.24	0.79–1.95	0.356	1.35	0.88–2.07	0.176
Invite	Never	159	66.3	1.00			1.00		
	Sometimes	157	53.2	0.62	0.43–0.91	0.013	0.65	0.46–0.93	0.018
	Always	110	51.2	0.71	0.45–1.14	0.160	0.73	0.48–1.11	0.144
Watch	Never	202	56.3	1.00					
	Sometimes	156	57.6	1.00	0.72–1.40	0.990			
	Always	68	57.6	1.02	0.64–1.63	0.942			
Remark	Never	222	60.3	1.00					
	Sometimes	139	53.3	1.05	0.75–1.48	0.777			
	Always	65	54.6	0.90	0.56–1.43	0.649			

OR: odds ratio; 95%CI: 95% confidence interval

Note: Only the variables presenting p values ≤ 0.20 in the crude analysis remained in the adjusted analysis.

## DISCUSSION

Adolescence is a critical moment for establishing physical activity (PA) habits; yet, the literature has depicted this life period with a gradual engagement decline across the various domains of PA^16^^,^^17^. Thus, our study targeted adolescents aged from 15 to 17 years. Active transportation to school (ATS) is an effective way of incorporating PA into daily activities and increasing adolescents’ overall PA levels^1^^,^^2^ – especially during transition from late adolescence to young adulthood, a critical period for decreasing PA levels^16^^–^^18^. Mandic et al.^19^, point that adolescents’ decision in adopting ATS may be influenced by several factors of personal, social, or environmental nature. Our research aimed to verify how sociodemographic factors (personal) and social support for PA practice (social) are associated with the use of ATS among adolescents enrolled in public schools in a large capital of Southern Brazil.

Regarding the prevalence of ATS in this research, 37% of the adolescents reported practicing this behavior. A recent systematic review on ATS among Brazilian adolescents identified a great variability in prevalence, ranging from 34.3% to 75.7%, depending on the evaluated study^2^. Most studies covered by this review reported a higher prevalence than that found in our research. However, only two of the studies included in this review involving national sampling presented data from adolescents from Curitiba^2^.

Regarding gender, we found that 21.5% of ATS is used by girls. Other studies^1^^–^^4^ also found a higher prevalence of active transportation among girls, but they are not unanimous, so these results should be interpreted with caution. Girls’ PA levels are lower than their peers in the context of leisure^18^, so transportation may be a way to increase girls’ PA. However, further research is required to investigate the prevalence of ATS considering gender. We should also consider that our research deals with ATS to and from school, while others deal only with one-way ATS. Validating instruments on active transportation for specific populations could help standardizing measures.

Studies have considered sociodemographic factors as possible influencers of ATS among adolescents^19^. Our results show association between these factors and ATS use only among girls, specially at the age of 16 – which may be related to the fact that older girls are more autonomous than younger girls. Further investigations may address the possibility of maximizing girls’ PA levels at the transport domain. Some studies show that older adolescents (both boys and girls) often use passive transport modes, such as car and motorcycle, even to nearby destinations^1^^,^^20^. These results suggest a need for greater efforts to understand factors that may influence the use of ATS among older adolescents, aiming to increase the use of this mode of commuting to school and other destinations^1^^,^^20^.

Conversely, this study found no association between ATS and sociodemographic factors such as parental schooling and socioeconomic status, deemed as important predictors of the different PA contexts (transportation and leisure)^16^^,^^20^^,^^21^. What motivates these results is still unclear, but a possibility is that higher parental schooling and socioeconomic status encounter different barriers to ATS, such as schedules or perception of safety^1^^–^^3^, regardless of the support for PA practice in other contexts.

Social support is associated with adolescents’ increased engagement in the different domains of PA^20^. The literature suggests that adolescents (boys and girls) report higher PA levels when their parents and friends provide greater support.^13^ Our study found that ATS was favored among boys who have parents that practice PA together with them, provide transportation for PA, and remark their good performance on it. We also observed that boys who have friends that practice PA with them are twice as likely to use ATS.

As for girls, having friends who practice PA with them was associated with ATS. Some studies state that the lack of company is the main barrier for girls to practice PA in leisure time^22^. This suggests that having friends who invite them may increase the odds of girls being active in the different contexts of PA (leisure and transportation). Our study corroborates this line by investigating the context of transportation.

Interestingly, the use of ATS was less likely among boys with encouraging parents and friends who watch their PA practice, and girls with friends who invite them to practice PA. This study does not explain what motivates such associations, but it reinforces the need for a more detailed research on how each social support characteristic can impact different types of PA practice among adolescents, possibly integrating quantitative and qualitative methods of research. Both genders presented associations related to social support from friends, differently from social support from parents, which was associated only in boys. This may be explained by a greater influence of parents for male children when it comes to practicing PA, enabling this finding to be a discussion topic for future studies investigating actions for gender equity.

When interpreting this study results, we should consider some limitations. First, due to the cross-sectional design, no causal relationship can be drawn from the results. Second, a self-reported questionnaire may lead participants to overestimate or underestimate the use of the transport modes at issue. The lack of knowledge regarding distance is a fundamental limitation. Further studies should consider including objective measures (GPS, for example) and subjective measures of transport behavior. Our study sample is formed only by students from public schools, which precludes the extrapolation of results to higher classes. However, the representative sample and statistical analyses ensure data interpretation for large populations of public schools – a key point in the field of interventions related to public health and prevention. This study also corroborates the investigation of sociodemographic and psychosocial factors for ATS among a representative sample of adolescents. We did not investigate the significant association between ATS and body mass index (BMI) and recommended levels of physical activity; however, we suggest that further studies evaluate this relationship for a greater clarification on ATS.

## CONCLUSION

The results show a 37.7% prevalence of active transportation to school (ATS): 16.2% among boys and 21.5% among girls. We found no association between sociodemographic factors and ATS, except age for girls. Social support for physical activity (PA) practice, from both parents and friends, was associated with ATS. For boys, ATS was associated with having parents who: practice PA together with them, provide transportation for PA, and remark their good performance on it; as well as having friends who practice PA together with them. For girls, ATS was solely associated with age and having friends who practice PA together with them.
