# Supplementary material for: Prevalence and factors associated with active transportation to school for adolescents
Source: Rev Saude Publica. 2020 Aug 5;54:78. doi: 10.11606/s1518-8787.2020054002078 (PMC7416599; doi:10.11606/s1518-8787.2020054002078)
Supplement: Translation

# Translation: Prevalência e fatores associados ao transporte ativo para a escola em adolescentes

**Authors:** Edina Maria de Camargo, Michael Pereira da Silva, Jorge Mota, Wagner de Campos

**Affiliations:** I CuritibaPR Brasil Universidade Federal do Paraná. Programa de Pós-Graduação em Educação Física. Curitiba, PR, Brasil; II Rio GrandeRS Brasil Universidade Federal do Rio Grande. Faculdade de Medicina. Rio Grande, RS, Brasil; III Porto Portugal Universidade do Porto. Faculdade de Desporto da Universidade do Porto. Departamento de Educação Física. Porto, Portugal; IV CuritibaPR Brasil Universidade Federal do Paraná. Departamento de Educação Física. Curitiba, PR, Brasil

**Keywords:** Adolescente, Caminhada, Atividade Motora, Fatores Socioeconômicos, Estudos Transversais

## Abstract

**OBJETIVO:**

Verificar a prevalência e os fatores associados ao transporte ativo para a escola em adolescentes brasileiros de escolas públicas.

**MÉTODOS:**

Estudo transversal, com amostra representativa de 1.984 adolescentes (55,9% meninas). Os fatores sociodemográficos incluídos foram sexo, idade, escolaridade dos pais e nível socioeconômico. Os fatores psicossociais incluídos foram apoio social dos pais e dos amigos para a atividade física. Foi considerado transporte ativo o ato de caminhar, pedalar ou usar skate no deslocamento para a escola. Modelos de regressão logística binária verificaram a associação dos fatores sociodemográficos e psicossociais ao transporte ativo para a escola adotando p < 0,05.

**RESULTADOS:**

A prevalência de transporte ativo para a escola foi de 37,7% (16,2% meninos e 21,5% meninas). Para meninos, o apoio social dos pais ao praticar atividade física junto (RC = 1,57; IC95% 1,09–2,25), transportar para a atividade física (RC = 1,56; IC95% 1,04–2,32) e comentar que o adolescente faz a atividade física bem (RC = 1,73; IC95% 1,08–2,76), assim como o apoio dos amigos ao praticar junto (RC = 2,23; IC95% 1,35–3,69), foram associados ao transporte ativo. Para meninas, a idade (RC = 1,43; IC95% 1,06–1,92) e ter amigos que praticam atividade física junto (RC = 1,48; IC95% 1,04–2,10) aumentaram as chances de transporte ativo para a escola.

**CONCLUSÃO:**

A idade e o apoio social para a prática de atividade física foram associados ao transporte ativo para a escola. Pais que praticam junto, transportam e comentam sobre a atividade aumentaram as chances para adolescentes meninos. O apoio social dos amigos para a atividade física aumentou as chances para ambos os sexos.

## INTRODUÇÃO

O transporte ativo para a escola (TAE) é uma maneira eficaz de aumentar os níveis gerais de atividade física (AF) em adolescentes^1^. Adolescentes envolvidos com TAE, como caminhar e andar de bicicleta, acumulam mais AF e apresentam menores riscos cardiometabólicos como sobrepeso e obesidade, diabetes e síndrome metabólica^2^^–^^4^. Apesar dos potenciais benefícios para a saúde, estudos relataram um declínio na proporção de crianças e adolescentes que usam TAE nas últimas décadas^2^^–^^4^, podendo contribuir para um declínio global da AF. Diante disso, a promoção do TAE tornou-se um importante tema presente em iniciativas internacionais que visam aumentar o nível de AF na população^3^^–^^6^.

Modelos ecológicos afirmam que os comportamentos de saúde, como o transporte ativo, são influenciados por vários fatores em múltiplos níveis, incluindo fatores psicossociais e ambientais^5^^,^^6^. Além disso, esses fatores podem variar de acordo com o sexo, idade, escolaridade dos pais e nível socioeconômico^5^^,^^6^. Como exemplo dessa influência, sabe-se que adolescentes que residem próximo à escola são mais propensos à adoção do TAE^1^^,^^2^; no entanto, mesmo para destinos mais próximos, os adolescentes mais velhos usam regularmente modos de transporte passivos, como carro e motocicleta^1^^,^^2^. Tais resultados indicam a necessidade de maiores esforços para o entendimento de fatores que podem influenciar o uso do TAE, visando aumentar esse tipo de deslocamento para a escola e para outros destinos^1^^,^^2^.

No Brasil, nos últimos anos, ocorreram tentativas de melhorar as características do ambiente urbano, podendo favorecer o uso de transporte ativo pela população, como a criação de ciclovias e ciclofaixas^7^. Iniciativas como essas têm impacto em médio e longo prazo, além de atingir um grande número de pessoas^8^. O entendimento da relação dos fatores sociodemográficos e psicossociais, em específico o apoio dos pais e amigos para prática da atividade física em seus diferentes contextos (lazer e transporte), pode fornecer informações de grande valia para as autoridades de saúde pública na implementação e promoção do TAE e, consequentemente, aumentar os níveis de AF. Diante disso, o objetivo deste estudo foi investigar a prevalência e os fatores associados ao transporte ativo para a escola em adolescentes brasileiros de escolas públicas.

## MÉTODOS

Trata-se de um estudo com delineamento transversal, realizado no ano de 2018, com amostra representativa de adolescentes de 15 a 17 anos de idade matriculados no ensino médio da rede estadual da cidade de Curitiba, Paraná, Brasil. O estudo seguiu as normas de pesquisa envolvendo seres humanos do Conselho Nacional de Saúde (resolução nº 466/2012), sendo aprovado pelo Comitê de Ética em Pesquisa da Universidade Federal do Paraná (CAAE: 98133218.8.0000.0102) e autorizado pelos pais ou responsáveis dos adolescentes pela assinatura do termo de consentimento livre e esclarecido (TCLE).

De acordo com o Censo Escolar de 2017, realizado pelo Instituto Nacional de Estudos e Pesquisas Educacionais Anísio Teixeira (Inep), havia naquele ano 53.760 adolescentes no ensino médio, de ambos os sexos, com idades entre 15 e 17 anos, nas escolas estaduais da rede de ensino público da cidade. Para o cálculo amostral, foi utilizado o software G*Power. Foi considerada uma razão de prevalências (RP) de 1,49 entre o apoio social (AS) e a prática de atividades físicas^9^, uma prevalência de transporte ativo de 50%^2^, um nível de confiança de 95% (α = 0,05) com um poder de 80% (β = 0,20) e um acréscimo de 30% para possíveis perdas e recusas, o que resultou em uma amostra necessária estimada de 965 meninos e 965 meninas, totalizando 1.930 adolescentes.

A amostragem partiu do processo de conglomerados, em três estágios. No primeiro, todas as escolas estaduais foram estratificadas de acordo com cada uma das nove regiões administrativas do município de Curitiba; no segundo, foi realizado um sorteio de duas escolas em cada uma das nove regiões administrativas; no terceiro, foi realizada uma seleção aleatória simples de uma turma de cada ano do ensino médio, de acordo com a quantidade de escolares necessária por sexo para determinada região administrativa do município. A seleção aleatória contemplou turmas do turno matutino e vespertino. Por fim, todos os alunos de cada turma foram convidados a participar do estudo.

No total foram convidados a participar do estudo 2.506 adolescentes. Os que não apresentaram o TCLE assinado pelos pais ou responsáveis (n = 100), que se negaram a participar do estudo ou que faltaram no dia da coleta (n = 56) não foram incluídos. Aqueles que informaram limitações físicas ou cognitivas que limitassem a prática de AF (n = 12) e que tinham 18 anos (n = 125) foram excluídos. Os que responderam aos questionários incorretamente (n = 229) foram considerados como perda amostral. Assim, a amostra analítica do estudo foi de 1.984 adolescentes. Análises de poder amostral realizadas *a posteriori* mostraram que essa amostra poderia identificar razões de prevalências estatisticamente significantes acima de RP = 1,28 como aumento do uso do transporte ativo para escola e abaixo de RP = 0,77 como menos propensos a utilizar transporte ativo para escola, considerando uma prevalência de 34% de adolescentes com baixo apoio social e que não realizam transporte ativo.

### Sexo, Idade, Escolaridade e Nível Socioeconômico

O sexo foi autorreportado (masculino ou feminino) e a idade calculada a partir da data de nascimento informada pelo adolescente e classificada em 15, 16 ou 17 anos. A escolaridade dos pais e responsáveis, bem como o nível socioeconômico, foram avaliados conforme a classificação da Associação Brasileira de Empresas de Pesquisa (ABEP)^10^. A escolaridade dos pais foi classificada em: até fundamental completo, ensino médio completo, ou ensino superior completo (foi perguntada a escolaridade do pai e da mãe e indicado quem era o chefe da família). O Critério de Classificação Econômica Brasil (CCEB)^10^ categoriza os estratos econômicos em classes A, B1, B2, C1, C2 e D-E, tendo como base os dados da Pesquisa Nacional por Amostra de Domicílios (PNAD). Para efeitos de análise, e com o objetivo de garantir comparabilidade com estudos semelhantes, o nível socioeconômico (NSE) foi classificado em três categorias: baixo (classes C+D+E), médio (classes B1+B2) ou alto (classes A1+A2).

### Apoio Social de Pais e Amigos

O apoio social de pais e amigos para a prática de AF foi mensurado por uma escala de 10 itens, a escala Asafa, que apresenta consistência interna satisfatória (pais: α ≥ 0,77 e índice de fidedignidade combinada [IFC] ≥ 0,83; amigos: α ≥ 0,87 e IFC ≥ 0,91)^11^. Os adolescentes informaram a frequência (nunca = 1, raramente = 2, frequentemente = 3, sempre = 4) com que os pais e amigos ofereciam algum tipo de apoio social para a prática de AF (incentivam, praticam, transportam, assistem, comentam, convidam) durante uma semana típica ou normal^11^, por meio das questões: “Com que frequência os seus pais: Incentivam você a praticar AF? Praticam AF com você? Transportam ou disponibilizam transporte para que você possa ir até o local onde pratica AF? Assistem você praticando AF? Comentam que você está praticando bem suas AF?” e “Com que frequência os seus amigos: Incentivam você a praticar AF? Praticam AF com você? Convidam você para praticar AF com eles? Assistem você praticando AF? Comentam que você está praticando bem suas AF?”

Para efeitos de análise, e com o objetivo de garantir comparabilidade com estudos semelhantes^12^^,^^13^, as opções de resposta “raramente” e “frequentemente” foram agrupadas e classificadas como “às vezes”. Assim, as categorias de frequência semanal de AS foram classificadas como nunca, às vezes ou sempre.

### Transporte Ativo para a Escola

Foi avaliado o modo de transporte usado para ir e voltar da escola durante uma semana típica (caminhada, bicicleta, skate, ônibus, van ou carro). Todos os estudantes que relataram caminhar ou andar de bicicleta ou skate para ir e voltar da escola foram classificados como “ativos” e os demais como “passivos”. Essa questão mostrou confiabilidade teste-reteste adequada (coeficiente de correlação intraclasse 0,90–0,95; p < 0,05) e tem sido utilizada em estudos semelhantes^14^^,^^15^.

### Análise dos Dados

Para evitar viés relacionado ao processo de seleção amostral com característica complexa (estratificação por conglomerado), as análises de associação foram corrigidas pelo delineamento complexo, utilizando o comando *complex sample* do software SPSS versão 23.0. Tal procedimento foi utilizado para assegurar que as estimativas reflitam os dados populacionais das unidades elementares da amostra.

As prevalências foram descritas pela distribuição de frequência relativa e absoluta. O teste qui-quadrado de Pearson foi utilizado para comparar proporções entre os sexos, e em seguida foi utilizado um *post hoc* para demonstrar quais categorias apresentaram maior diferença. Foi utilizada a regressão logística binária de forma bruta e ajustada para verificar a associação dos fatores demográficos e apoio social de pais e amigos com o TAE. Pelo critério *stepwise*, foi adotado o valor de p ≤ 0,20 para entrada da variável no modelo ajustado. Todas as análises foram realizadas separadamente para cada sexo e foi adotado um nível de significância de 5%.

## RESULTADOS

A amostra foi composta por 1.984 adolescentes, sendo 55,9% do sexo feminino. Desses, somente 748 (37,7%) relataram utilizar transporte ativo para a escola em uma semana habitual, sendo 16,2% meninos e 21,5% meninas. Nas tabelas 1 e 2, os dados foram estratificados de acordo com o sexo.

**Tabela 1:** Idade, escolaridade dos pais e nível socioeconômico (NSE) dos adolescentes de acordo com o sexo (n = 1.984).

		Masculino (n = 875; 44,1%)		Feminino (n = 1.109; 55,9%)		p^c^	Total	
		n	%	n	%		n	%
Idade	15 anos	261	41,8	363	58,2	0,124	624	100
	16 anos	317	44,3	399	55,7		716	
	17 anos	297	46,1	347	53,9		644	
Escolaridade do pai	Até fundamental completo	238^a^	38,1	387^a^	61,9	0,003	625	100
	Ensino médio completo	405^b^	47,2	453^b^	52,8		858	
	Ensino superior completo	232^b^	46,3	269^b^	53,7		501	
Escolaridade da mãe	Até fundamental completo	245^a^	39,0	384^a^	61,0	0,006	629	100
	Ensino médio completo	398^b^	46,3	461^b^	53,7		859	
	Ensino superior completo	232^b^	46,8	264^b^	53,2		496	
NSE	Baixo	140	40,0	210	60,0	0,005	350	100
	Médio	538^a^,^b^	43,4	702^a^,^b^	56,6		1.240	
	Alto	197	50,0	197	50,0		394	

a,b Diferem significativamente entre si; *post hoc* de *Bonferroni*.

c Teste qui-quadrado.

**Tabela 2:** Prevalência de apoio social de pais e amigos entre os adolescentes de acordo com o sexo (n = 1.984).

			Masculino (n = 875; 44,1%)		Feminino (n = 1.109; 55,9%)		p^c^	Total	
			n	%	n	%		n	%
Apoio social dos pais									
	Incentivam	Nunca	170	41,5	240	58,5	0,113	410	100
		Às vezes	473	43,9	604	56,1		1.077	
		Sempre	232	46,7	265	53,3		497	
	Praticam	Nunca	322	41,4	455	58,6	0,027	777	100
		Às vezes	434	45,4	530	55,0		964	
		Sempre	119	49,0	124	51,0		243	
	Transportam	Nunca	425	44,0	541	56,0	0,513	966	100
		Às vezes	262	42,6	353	57,4		615	
		Sempre	188	46,7	215	53,3		403	
	Assistem	Nunca	370	42,5	500	57,5	0,166	870	100
		Às vezes	365	44,8	450	55,2		815	
		Sempre	140	46,8	159	53,2		299	
	Comentam	Nunca	324	41,9	449	58,1	0,621	773	100
		Às vezes	341	47,6	375	52,4		716	
		Sempre	210	42,4	285	57,6		495	
Apoio social dos amigos									
	Incentivam	Nunca	324	42,1	445	57,9	0,240	769	100
		Às vezes	355	45,5	426	54,5		781	
		Sempre	196	45,2	238	54,8		434	
	Praticam	Nunca	229^a^	39,7	348^a^	60,3	0,005	577	100
		Às vezes	370^a^,^b^	44,6	460^a^,^b^	55,4		830	
		Sempre	276^b^	47,8	301^b^	52,2		577	
	Convidam	Nunca	243^a^	39,7	376^a^	60,7	0,015	619	100
		Às vezes	381^b^	46,4	440^b^	53,6		821	
		Sempre	251^a^,^b^	46,1	293^a^,^b^	53,9		544	
	Assistem	Nunca	421	44,7	520	55,3	0,811	941	100
		Às vezes	309	43,0	410	57,0		719	
		Sempre	145	44,8	179	55,2		324	
	Comentam	Nunca	425	42,9	566	57,1	0,462	991	100
		Às vezes	308	45,9	363	54,1		671	
		Sempre	142	44,1	180	55,9		322	

a,b Diferem significativamente entre si; *post hoc* de *Bonferroni*.

c Teste qui-quadrado.

As associações dos fatores sociodemográficos e do apoio social com o TAE para meninos podem ser verificadas na Tabela 3. Com relação aos fatores sociodemográficos, nenhuma das variáveis sociodemográficas esteve associada com o TAE. Já quanto ao apoio social dos pais, os resultados apresentados na análise ajustada identificaram que meninos cujos pais incentivam a prática de AF foram menos propensos a realizar o transporte ativo (às vezes: razão de chances [RC] = 0,64; IC95% 0,42–0,96; sempre: RC = 0,58; IC95% 0,36–0,95). Por outro lado, meninos cujos pais às vezes praticam AF junto (RC = 1,57; IC95% 1,09–2,25) e que sempre oferecem transporte para a prática de AF (RC = 1,56; IC95% 1,04–2,32), assim como pais que sempre comentam que os filhos fazem bem a AF (RC = 1,73; IC95% 1,08–2,76) usaram mais o TAE.

**Tabela 3:** Associação bruta e ajustada dos fatores sociodemográficos e do apoio social de pais e amigos com o transporte ativo para a escola em meninos (n = 875).

Fatores sociodemográficos		Bruta					Ajustada		
		n	%	RC	IC95%	p	RC	IC95%	p
Idade	15 anos	98	44,3	1,00					
	16 anos	129	43,1	1,14	0,81–1,60	0,441			
	17 anos	95	41,7	0,78	0,55–1,11	0,169			
Escolaridade do pai	Até fundamental completo	151	38,8	1					
	Ensino médio completo	251	47,8	1,13	0,79–1,61	0,508			
	Ensino superior completo	151	46,9	1,02	0,67–1,55	0,924			
Escolaridade da mãe	Até fundamental completo	96	37,6	1,00					
	Ensino médio completo	146	45,8	0,87	0,61–1,23	0,422			
	Ensino superior completo	80	46,0	0,81	0,54–1,23	0,321			
NSE	Baixo	49	37,4	1,00					
	Médio	192	42,0	1,03	0,70–1,52	0,880			
	Alto	81	50,6	1,30	0,83–2,03	0,256			

Apoio social dos pais		n	%	RC	IC95%	p	RC	IC95%	p
Incentivam	Nunca	67	38,7	1,00			1,00		
	Às vezes	169	43,4	0,67	0,45–1,00	0,051	0,64	0,42–0,96	0,033
	Sempre	86	46,2	0,60	0,37–0,98	0,039	0,58	0,36–0,95	0,030
Praticam	Nunca	99	35,7	1,00			1,00		
	Às vezes	178	46,8	1,49	1,05–2,10	0,026	1,57	1,09–2,25	0,015
	Sempre	45	49,5	1,19	0,70–2,03	0,522	1,23	0,72–2,09	0,443
Transportam	Nunca	140	38,5	1,00			1,00		
	Às vezes	95	42,4	1,06	0,75–1,51	0,735	1,03	0,72–1,48	0,869
	Sempre	87	54,4	1,64	1,11–2,41	0,013	1,56	1,04–2,32	0,030
Assistem	Nunca	130	40,5	1,00					
	Às vezes	139	43,6	0,83	0,57–1,21	0,322			
	Sempre	53	49,1	0,74	0,44–1,23	0,247			
Comentam	Nunca	99	36,4	1,00			1,00		
	Às vezes	134	47,5	1,55	1,04–2,32	0,031	1,39	0,93–2,05	0,105
	Sempre	89	45,9	1,72	1,09–2,73	0,020	1,73	1,08–2,76	0,023

Apoio social dos amigos		n	%	RC	IC95%	p	RC	IC95%	p
Incentivam	Nunca	117	40,1	1,00					
	Às vezes	137	46,3	0,93	0,64–1,34	0,687			
	Sempre	68	42,5	0,65	0,40–1,06	0,082			
Praticam	Nunca	70	34,8	1,00			1,00		
	Às vezes	140	43,5	1,54	1,00–2,39	0,050	1,50	0,97–2,30	0,066
	Sempre	112	49,8	1,97	1,16–3,33	0,011	2,23	1,35–3,69	0,002
Convidam	Nunca	81	33,8	1,00					
	Às vezes	138	46,8	1,03	0,65–1,58	0,954			
	Sempre	103	48,4	1,40	0,82–2,38	0,222			
Assistem	Nunca	157	43,7	1,00			1,00		
	Às vezes	115	42,4	0,70	0,47–1,04	0,076	0,63	0,42–0,95	0,026
	Sempre	50	42,4	0,53	0,30–0,93	0,026	0,45	0,25–0,79	0,006
Comentam	Nunca	146	39,7	1,00					
	Às vezes	122	46,7	1,30	0,90–1,88	0,165			
	Sempre	54	45,4	1,31	0,78–2,19	0,304			

RC: razão de chances; IC95%: intervalo de confiança de 95%; NSE: nível socioeconômico

Nota: Permaneceram na análise ajustada somente as variáveis que apresentaram valores de p ≤ 0,20 na análise bruta.

Com relação ao apoio social dos amigos, meninos que indicaram ter amigos que sempre praticam AF apresentaram maiores chances de realizar o TAE (RC = 2,23; IC95% 1,35–3,69). Além disso, meninos que têm amigos que os assistem realizando AF foram menos propensos a realizar o TAE (às vezes: RC = 0,63; IC95% 0,42–0,95; sempre: RC = 0,45; IC95% 0,25–0,79).

A Tabela 4 apresenta as associações dos fatores sociodemográficos e apoio social com o TAE para meninas. Dentre as variáveis sociodemográficas avaliadas, apenas a idade apresentou-se significativa no modelo ajustado, indicando que meninas com 16 anos foram mais propensas a realizar o TAE (RC = 1,43; IC95% 1,06–1,92). Quanto às variáveis relacionadas ao apoio social para a AF, apenas as referentes ao AS proveniente dos amigos permaneceram associadas no modelo ajustado, indicando que meninas que têm amigos que às vezes praticam AF junto com elas possuem mais chances de realizar o TAE quando comparado com aquelas que nunca recebem esse tipo de apoio (RC = 1,48; IC95% 1,04–2,10). Em contrapartida, meninas que têm amigos que às vezes as convidam para praticar AF foram menos propensas a realizar o TAE (RC = 0,65; IC95% 0,46–0,93).

**Tabela 4:** Associação bruta e ajustada dos fatores sociodemográficos e do apoio social de pais e amigos com o transporte ativo para a escola em meninas (n = 1.109).

Fatores sociodemográficos		Bruta					Ajustada		
		n	%	RC	IC95%	p	RC	IC95%	p
Idade	15 anos	123	55,7	1,00			1,00		
	16 anos	170	56,9	1,45	1,08–1,95	0,014	1,43	1,06–1,92	0,018
	17 anos	133	58,3	1,21	0,89–1,65	0,218	1,17	0,86–1,60	0,317
Escolaridade do pai	Até fundamental completo	238	61,2	1,00					
	Ensino médio completo	274	52,2	1,13	0,84–1,53	0,418			
	Ensino superior completo	171	53,1	1,03	0,71–1,48	0,854			
Escolaridade da mãe	Até fundamental completo	159	62,4	1,00					
	Ensino médio completo	173	54,2	0,82	0,60–1,10	0,182			
	Ensino superior completo	94	54,0	0,77	0,54–1,11	0,156			
NSE	Baixo	82	62,6	1,00					
	Médio	265	58,0	0,95	0,70–1,30	0,734			
	Alto	79	49,4	1,05	0,70–1,56	0,828			

Apoio social dos pais		n	%	RC	IC95%	p	RC	IC95%	p
Incentivam	Nunca	106	61,3	1,00					
	Às vezes	220	56,6	0,70	0,50–0,98	0,040			
	Sempre	100	53,8	0,80	0,52–1,23	0,314			
Praticam	Nunca	178	64,3	1,00					
	Às vezes	202	53,2	1,04	0,77–1,40	0,809			
	Sempre	46	50,5	1,03	0,63–1,66	0,921			
Transportam	Nunca	224	61,5	1,00					
	Às vezes	129	57,6	0,79	0,58–1,07	0,124			
	Sempre	73	45,6	0,71	0,48–1,04	0,076			
Assistem	Nunca	191	59,5	1,00					
	Às vezes	180	56,4	1,20	0,87–1,66	0,258			
	Sempre	55	50,9	0,98	0,62–1,54	0,919			
Comentam	Nunca	173	63,6	1,00					
	Às vezes	148	52,5	1,18	0,84–1,66	0,340			
	Sempre	105	54,1	1,08	0,72–1,61	0,722			

Apoio social dos amigos		n	%	RC	IC95%	p	RC	IC95%	p
Incentivam	Nunca	175	59,9	1,00					
	Às vezes	159	53,7	0,98	0,70–1,37	0,908			
	Sempre	92	57,5	1,09	0,72–1,66	0,683			
Praticam	Nunca	131	65,2	1,00			1,00		
	Às vezes	182	56,5	1,39	0,96–2,01	0,084	1,48	1,04–2,10	0,030
	Sempre	113	50,2	1,24	0,79–1,95	0,356	1,35	0,88–2,07	0,176
Convidam	Nunca	159	66,3	1,00			1,00		
	Às vezes	157	53,2	0,62	0,43–0,91	0,013	0,65	0,46–0,93	0,018
	Sempre	110	51,2	0,71	0,45–1,14	0,160	0,73	0,48–1,11	0,144
Assistem	Nunca	202	56,3	1,00					
	Às vezes	156	57,6	1,00	0,72–1,40	0,990			
	Sempre	68	57,6	1,02	0,64–1,63	0,942			
Comentam	Nunca	222	60,3	1,00					
	Às vezes	139	53,3	1,05	0,75–1,48	0,777			
	Sempre	65	54,6	0,90	0,56–1,43	0,649			

RC: razão de chances; IC95%: intervalo de confiança de 95%; NSE: nível socioeconômico

Nota: Permaneceram na análise ajustada somente as variáveis que apresentaram valores de p ≤ 0,20 na análise bruta.

## DISCUSSÃO

A adolescência apresenta-se como um importante momento para a consolidação dos hábitos de AF; de fato, a literatura vem demonstrando essa fase da vida como um período de declínio gradual do engajamento nos diversos domínios da AF^16^^,^^17^. Por esse motivo, este estudo buscou investigar adolescentes com idade entre 15 e 17 anos. O TAE é uma maneira eficaz de incorporar AF nas atividades diárias e aumentar os níveis gerais de AF^1^^,^^2^ de adolescentes, em especial na transição do final da adolescência para a idade adulta jovem, pois esse período é considerado crítico e associado à diminuição da AF^16^^–^^18^. Mandic et al.^19^ apontam que diversos fatores têm potencial de influenciar a decisão dos adolescentes para a adoção do TAE, classificando-os entre os de caráter pessoal, social e ambiental. O presente trabalho teve como objetivo verificar como características sociodemográficas (pessoais) e de apoio social à prática da AF (sociais) podem associar-se com a realização do TAE por adolescentes matriculados em escolas públicas de uma grande capital do sul do Brasil.

Com relação à prevalência do TAE nesta investigação, 37% dos adolescentes indicaram ter esse tipo de comportamento. Em recente revisão sistemática sobre o TAE em adolescentes brasileiros, os autores identificaram uma grande variabilidade nas prevalências, mais especificamente de 34,3% a 75,7%, dependendo do estudo avaliado^2^. Contudo, boa parte dos trabalhos incluídos nessa revisão apresentaram prevalências superiores à encontrada na presente investigação. Por outro lado, é importante ressaltar que somente com dois estudos incluídos nessa revisão envolvendo amostragem nacional apresentavam dados de adolescentes da cidade de Curitiba^2^.

Em relação ao sexo, o presente estudo mostrou que 21,5% do TAE é realizado por meninas. Outros estudos^1^^–^^4^ também encontraram uma maior prevalência de meninas no contexto do transporte, porém é preciso ter cautela na interpretação desses resultados, uma vez que os estudos não são unânimes. Entretanto, uma vez que no contexto do lazer os níveis de AF de meninas são inferiores a seus pares^18^, o transporte pode ser uma forma de promover AF no sexo feminino. Contudo, mais pesquisas que investiguem a prevalência do TAE considerando o sexo são necessárias. Ademais, deve-se levar em consideração que este trabalho considera o TAE no ir e vir da escola, enquanto outros consideram o TAE apenas para uma direção. A validação de instrumentos sobre transporte ativo para populações específicas poderia contribuir na padronização das medidas.

Fatores sociodemográficos têm sido considerados como possíveis influenciadores do TAE em adolescentes^19^. Nesta investigação, esses fatores apresentaram-se associados ao TAE apenas para as meninas; mais especificamente, a idade de 16 anos foi um importante preditor da realização do TAE para elas – o que pode ser influenciado pelo fato de as meninas mais velhas serem mais autônomas do que as mais novas. A possibilidade de meninas maximizarem o nível de AF com o domínio transporte pode ser tema de estudo em futuras investigações. Alguns trabalhos mostram que, mesmo para destinos mais próximos, os adolescentes mais velhos (tanto meninos quanto meninas) usam regularmente modos de transporte passivos, como carro e motocicleta^1^^,^^20^. Tais resultados indicam a necessidade de maiores esforços para o entendimento de fatores que podem influenciar o uso de TAE em adolescentes mais velhos, visando aumentar esse tipo de deslocamento para a escola e para outros destinos^1^^,^^20^.

Por outro lado, fatores sociodemográficos como a escolaridade dos pais e o nível socioeconômico, que são apresentados como importantes preditores dos diferentes contextos da AF (transporte e lazer)^16^^,^^20^^,^^21^, não estiveram associados ao TAE neste estudo em nenhum dos sexos. As razões para a ocorrência desses resultados não são claras, mas é possível que a escolaridade dos pais e o nível socioeconômico mais elevado considerem outras barreiras para o TAE, tais como os horários ou a percepção de segurança^1^^–^^3^, independentemente do suporte para a prática de AF em outros contextos.

O apoio social apresenta-se associado com o maior engajamento de adolescentes com a AF em seus diferentes domínios^20^. A literatura indica que, quando pais e amigos fornecem maior apoio, os adolescentes (meninos e meninas) passam a referir maiores níveis de AF^13^. O presente estudo verificou que, para os meninos, ter pais que praticam AF junto com eles, que fornecem transporte para a AF e que comentam que eles estão fazendo bem a atividade foram importantes favorecedores do TAE. Adicionalmente, meninos que tinham amigos que praticavam AF com eles apresentaram duas vezes mais chances de realizar TAE.

Já para as meninas, ter amigos que praticam AF com elas associou-se ao TAE. Alguns estudos mencionam que para meninas a principal barreira para a prática de AF no lazer é a falta de companhia^22^. Isso sugere que ter amigos que as convidam pode aumentar as chances de as meninas serem ativas nos diferentes contextos da AF (lazer e transporte). O presente estudo colabora com essa linha ao investigar o contexto do transporte.

Curiosamente, meninos com pais que incentivam e amigos que assistem à prática de AF e meninas com amigos que as convidam para a prática de AF foram menos propensos à realização do TAE. Este estudo não apresenta explicações plausíveis para essas associações, mas reforça a necessidade de uma investigação mais detalhada sobre como cada característica de apoio social pode impactar diferentes tipos de prática de AF nesses adolescentes, possivelmente incluindo uma interação entre metodologias de investigações quantitativas e qualitativas. Em síntese, as associações referentes ao apoio social de amigos ocorreram em ambos os sexos, diferentemente do apoio social dos pais, que esteve associado somente no sexo masculino. É possível que esses dados sejam resultados de uma maior influência dos pais para filhos do sexo masculino quando o assunto é praticar AF, o que torna esse resultado tema de discussão para futuros estudos que busquem investigar ações para a equidade entre os sexos.

Alguns pontos devem ser considerados na interpretação dos resultados deste estudo. A primeira limitação é que nenhuma relação causal pode ser desenhada devido ao delineamento transversal. Em segundo lugar, um questionário autorrelatado pode levar os participantes a superestimar ou subestimar o uso de modos de transportes questionados. O não conhecimento da distância torna-se uma limitação fundamental. Futuros estudos devem considerar a inclusão de medidas objetivas (usando GPS, por exemplo) somadas a medidas subjetivas do comportamento de transporte. O fato de a amostra ser formada apenas por estudantes da rede pública impossibilita a extrapolação dos resultados para classes mais elevadas. Entretanto, a amostra representativa e as análises estatísticas asseguram uma interpretação dos dados para grandes populações de escolas da rede pública, um ponto importante no domínio de intervenções relacionadas à saúde pública e preventiva. Ademais, o estudo colabora na investigação dos fatores sociodemográficos e psicossociais para o TAE em uma amostra representativa de adolescentes. Apesar de não ter investigado a relação significativa do TAE com o índice de massa corporal (IMC) e níveis recomendados de atividade física, os autores sugerem que futuros estudos avaliem essa relação, com o objetivo de agregar maiores esclarecimentos sobre o TAE.

## CONCLUSÃO

Os resultados indicam uma prevalência de TAE de 37,7%, sendo 16,2% entre meninos e 21,5% entre meninas. Os fatores sociodemográficos não foram associados com o TAE, exceto a idade para as meninas. O apoio social para a prática de AF, tanto de pais quanto de amigos, associou-se ao TAE. Para os meninos, ter pais que praticam AF junto, fornecem transporte para a AF e comentam que o adolescente faz bem a AF apresentou associação com o TAE, assim como ter amigos que praticam AF junto. Para as meninas, apenas a idade e ter amigos que praticam AF junto se associaram ao TAE.
